# The Design of Terahertz Monolithic Integrated Frequency Multipliers Based on Gallium Arsenide Material

**DOI:** 10.3390/mi11030336

**Published:** 2020-03-24

**Authors:** Jin Meng, Luwei Qi, Xiaoyu Liu, Jingtao Zhou, Dehai Zhang, Zhi Jin

**Affiliations:** 1 Key Lab. of Microwave Remote Sensing, National Space Science Center, Chinese Academy of Sciences, Beijing 100190, China; qi7luwei@163.com (L.Q.); zhangdehai@nssc.ac.cn (D.Z.); 2 Institute of Microeletronics, Chinese Academy of Sciences, Beijing 100019, China; liusiyu@nssc.ac.cn (X.L.); zhoujingtao@ime.ac.cn (J.Z.); jinzhi@ime.ac.cn (Z.J.); 3 School of Electronic Engineering, University of Electronic Science and Technology of China, Chengdu 611731, China

**Keywords:** Schottky diode, terahertz monolithic integrated circuit, field-circuit method

## Abstract

A global design method for a terahertz monolithic integrated frequency multiplier is proposed. Compared with a traditional independent design, the method in this paper adopts overall optimization and combines the device with the circuit design. The advantage is that it provides a customized design for frequency multipliers according to specifications. On the basis of the gallium arsenide process of the Institute of Microelectronics, Chinese Academy of Sciences, two types of Schottky diodes have been developed to meet the needs of different designs. On the one hand, a Schottky diode with a 3 μm junction’s diameter was used in the design of the 200 GHz balanced doubler and, on the other hand, a diode with a 5 μm diameter was used in the 215 GHz unbalanced tripler. The measured results indicated that the output power of the doubler was more than 250 μW at 180~218 GHz, and the maximum was 950 μW at 198 GHz when driven with 12.3 mW, whereas that of the tripler was above 5 mW at 210~218 GHz and the maximum exceeded 10 mW. Such frequency multiplier sources could be widely used in terahertz imaging, radiometers, and so on.

## 1. Introduction

In recent decades, terahertz technology has been used for a variety of applications such as communication, earth atmospheric sensing, space astrophysics, etc. [1,2,3,4,5]. Furthermore, advances in terahertz sources and detectors have facilitated the development of these terahertz applications. The generation of a terahertz signal generally uses solid-state frequency multipliers based on Schottky diodes in order to reduce mass, volume and, complexity.

All solid-state sources above 1 THz, which produce tens of microwatts of output power, have been realized by some overseas leading research institutes such as Jet Propulsion Laboratory (JPL). To increase the cutoff frequency and reduce the transmission loss, several competing semiconductor technologies such as frameless membrane and substrate transfer technique have been used in terahertz monolithic integrated circuit (TMIC) design [6,7,8,9] As compared with the TMIC, the studies on frequency multipliers at terahertz wave range, in China, have mainly focused on a hybrid integrated circuit (HIC) with discrete Schottky diodes [10,11,12,13,14]. However, it is difficult to design frequency multipliers because there are limited kinds of commercial discrete diode chips available.

A global design method for a TMIC is proposed in this paper, including the device design, technological process, circuit design, etc. The details of steps mentioned above are discussed in the following sections.

## 2. Materials and Device 

**Figure 1:**
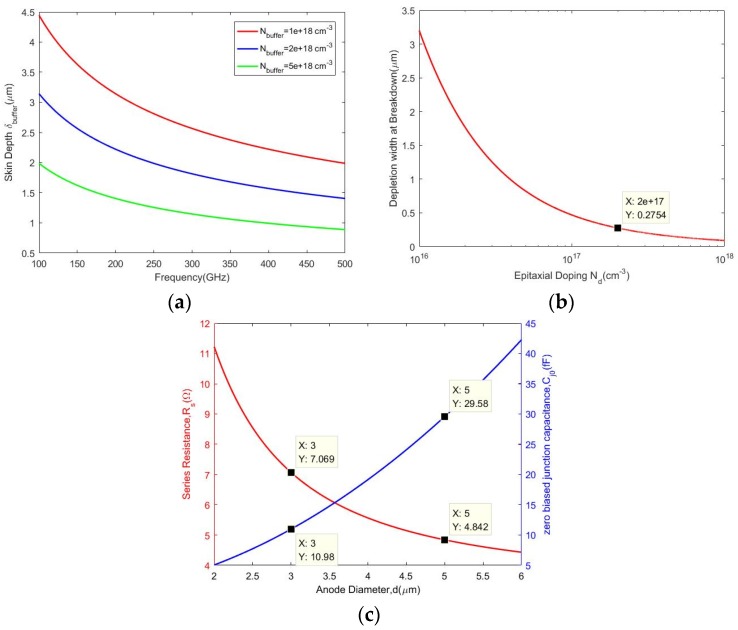
The simulation characteristics of the Schottky diode based on gallium arsenide material. (**a**) Skin depth of the buffer layer varies with frequency at different doping concentrations; (**b**) The thickness of the depletion layer varies with doping concentration of epitaxial layer; (**c**) Parameters of the Schottky junction vary with the anode diameter.

The general structure of most Schottky devices is similar. An n epitaxial layer approximately a few tenths of micron thick is grown on top of the high-conductivity buffer layer, which has a thickness of a few microns grown on top of the substrate. In addition, the contact of the metal to the semi-conducting layer forms the Schottky junction. First, it is necessary to determine the dimension of the diode, especially the thickness of the epitaxial and buffer layers. Figure 1a shows the relationship of skin depth in the buffer layer with the frequency at different doping concentrations. The doping concentration of the buffer layer, in this paper, is about 5 × 10^18^ cm^−3^, hence the thickness of the buffer layer is selected as 1.5 μm with the aim of working at approximately 200 GHz. With regard to the epitaxial layer, the thickness needs to be greater than the depletion width when the voltage equals breakdown. As shown in Figure 1b, when the doping concentration is 2 × 10^17^ cm^−3^, the thickness of the lightly doped n-type GaAs layer is 0.3 μm. Secondly, to adapt the design of different frequency multipliers, there are two junction sizes, one with a diameter of 3 μm, and the other with a diameter of 5 μm. For a Schottky diode, the parameters mainly include zero bias junction capacitance (*C_j_*_0_), series resistance (*Rs*), ideal factor (*n*), and reverse saturation current (*Is*). These parameters can be expressed as [15,16,17]:

(1) $$c_{j0}=A_{a}\cdot \gamma \left(0\right)\cdot \sqrt{\frac{q\cdot N_{D}\cdot \varepsilon_{s}}{2\cdot V_{bi}}}$$

(2) $$R_{s}\left(V_{j},f\right)=R_{e}\left(V_{j},f\right)+R_{p}\left(f\right)+R_{o}\left(f\right)$$

(3) $$I_{s}=A_{a}A^{**}T^{2}\left(e^{\frac{-q\emptyset_{b}}{kT}}\right)$$

(4) $$n={\left(kT\left(\frac{tanh\left(\frac{E_{00}}{kT}\right)}{E_{00}}-\frac{1}{2E_{B}}\right)\right)}^{-1}$$

where *A*_a_ is the anode area, *N*_D_ is the epitaxial layer doping, *V*_bi_ is the built-in voltage, *ε*_s_ is the gallium arsenide dielectric permittivity, *R*_e_ is the epitaxial layer resistance, *R*_p_ is the buffer spreading resistance, *R*_o_ is the ohmic resistance, A** is the effective Richardson constant, *Φ*_b_ is barrier height, *E*_B_ is the band bending, and *kT*/*q* equals 25.8 mV. In addition, *γ*(0) is a correction term and can be expressed as:

(5) $$\gamma \left(0\right)=1+\frac{1.5\cdot \sqrt{\frac{2\cdot \varepsilon_{s}\cdot V_{bi}}{q\cdot N_{D}}}}{r_{a}}$$

*E*_00_ is defined as material constant, and can be written as,

(6) $$E_{00}=18.5\times 10^{-12}\sqrt{\frac{N_{d}}{m_{e}\varepsilon_{r}}}$$

where *m*_e_ is the relative effective mass of electron and *ε*_r_ is the relative dielectric constant.

Actually, *C_j_*_0_ and *Rs* play a more important role in the circuit simulation. As depicted in Figure 1c, the zero bias junction capacitance (*C_j_*_0_) and series resistance (*Rs*) of the Schottky junction vary with the anode diameter. It is found that the value of *Rs* declines with the rise of anode diameter, while *C_j_*_0_ changes in an opposite way. 

**Figure 2:**
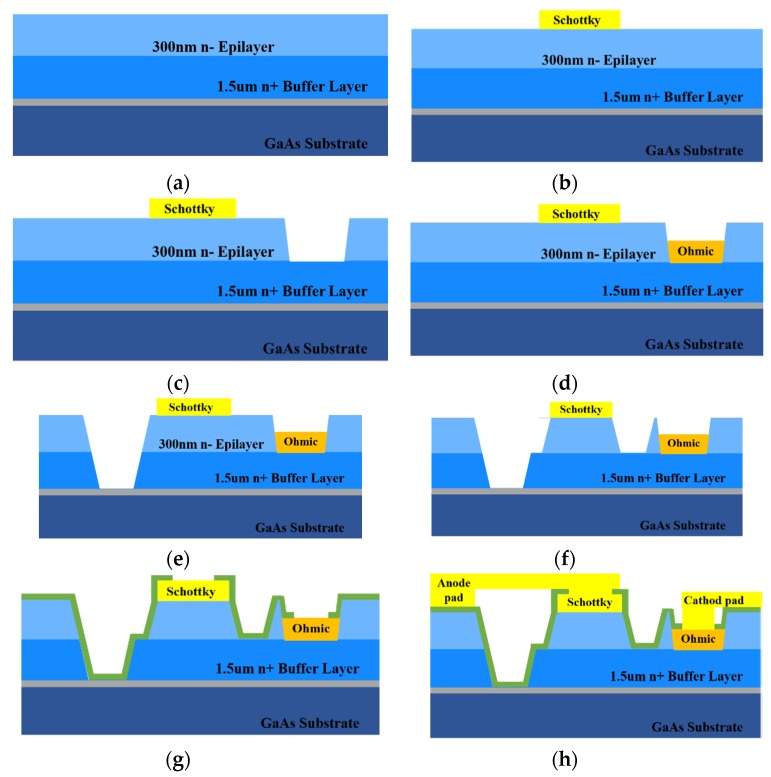
The fabrication process of the Schottky diode based on the gallium arsenide process of the Institute of Microelectronics, Chinese Academy of Sciences. (**a**) Epitaxial wafer preparation, (**b**) Schottky metal deposition, (**c**) ohmic definition, (**d**) ohmic contact fabrication, (**e**) Mesa separation, (**f**) Schottky terminal structure formation, (**g**) passivation, (**h**) Interconnect metal and airbridge electroplating.

The fabrication process is shown in Figure 2. The initial material is a semi-insulating 360 μm gallium arsenide substrate and epitaxial layers with different doping concentrations. Some key steps are as follows: First, Ti/Pt/Au metal films are evaporated to form the Schottky contacts. Then, ohmic definition is realized by using wet etching, and Ni/Ge/Au are deposited on the n+ gallium arsenide buffer layer by E-beam evaporation to form cathode ohmic contacts. Subsequently, a selective gallium arsenide wet etching is used to define the device mesas and a Schottky junction terminal structure is formed. Furthermore, samples are passivated with 300 nm SiO_2_ by plasma enhanced chemical vapor deposition (PECVD), and the dielectric layer existing on top of the metal contacts is removed via SF6 dry etching. The next process is completed with an interconnection metal electroplate to form an air bridge. Finally, the gallium arsenide substrate is thinned from the backside to the desired thickness (25~35 μm) by lapping and polishing. On the basis of the gallium arsenide process of the Institute of Microelectronics, Chinese Academy of Sciences, a batch of diodes are developed and an S parameter measurement is done at low frequency. At the same time, the diode’s equivalent circuit is built in software, and the parameters are extracted by using S-parameter curve fitting [18,19]. Table 1 shows the simulated and measured parameters of the Schottky diodes with different diameters. It is easy to find that the values have good consistency.

## 3. Circuit Design

To improve the accuracy of the simulation and reduce the complexity of the model, a global field-circuit method is applied to the design process, and therefore the frequency multiplier is divided into the following two parts: A linear network, which is analyzed using an Ansys High Frequency Structure Simulator (HFSS, Ansys, Inc., Canonsburg, Pennsylvania, USA) and in consideration of the parasitic effects, the nonlinear behavior of the diode is solved using a Agilent Advanced Design Simulator (ADS, Keysight technologies, Inc., Santa Rosa, CA, USA) [20,21,22].

**Figure 3:**
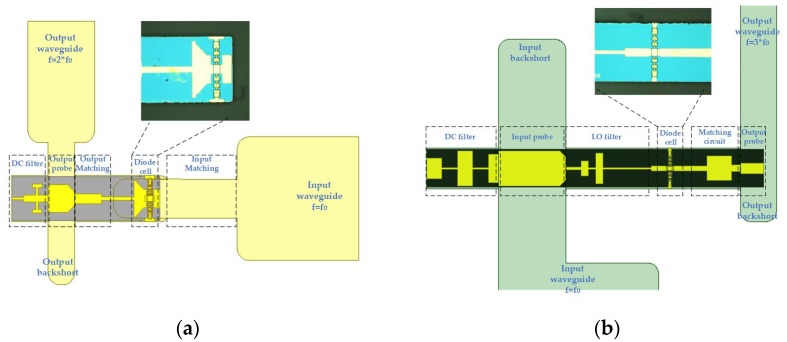
The overall circuit model in the high frequency structure simulator. (**a**) The structure of wideband doubler; (**b**) The structure of unbalanced tripler. The images of the diode cell in monolithic integrated circuit are added through a microscope.

Regarding the balanced doubler, the design is based on a monolithic integrated circuit and a diode with a diameter of 3 μm is used. Generally, the function of a doubler is to convert a pump microwave signal to its second harmonic based on the nonlinear characteristics of the diode junction, and the diode array in the doubler adopts an anti-series type to suppress the odd harmonics [23,24,25]. Figure 3a shows the overall structure of the 200 GHz doubler, and it mainly includes input waveguide, input matching, diode cell, etc. The incident signal with dominant mode is fed by the WR8 (2032 × 1016 μm) rectangular waveguide with reduced height to 320 μm, whereas the second harmonics is WR4 (1092 × 546 μm) with reduced height to 220 μm. In the upper area of Figure 3a, the photograph of the monolithic integrated circuit based on 25 μm gallium arsenide substrate is shown under a microscope.

**Figure 4:**
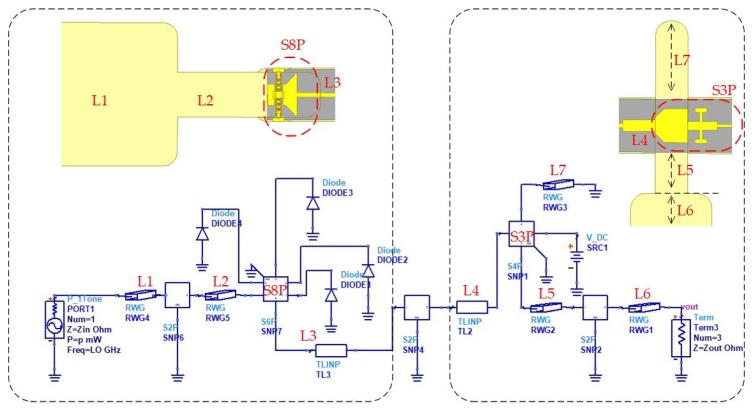
Design of the 200 GHz monolithic integrated doubler based on the field-circuit method (L1 = 0.7 mm, L2 = 0.57 mm, L3 = 0.1 mm, L4 = 0.22 mm, L5 = 0.27 mm, L6 = 0.15 mm, and L7 = 0.52 mm).

The equivalent linear and nonlinear models of the doubler are shown in Figure 4. Actually, a one-to-one correspondence exists between the elements of two circuits. The first step in the design of a linear circuit is to obtain the optimum impedances of the diode at fundamental and second harmonic using source and load-pull under ideal conditions [26,27]. The final calculation results show that the impedance of source and load are equal to 58- j26Ω and 20- j17Ω, respectively. Then, the Schottky junction is replaced by impedances in the linear simulation, which is also known as field analysis using the finite element method. The signal (TE10 mode) is coupled through waveguide-microstrip structure, and the length of the reduced height waveguide and location of the input back-short are optimized to achieve the return loss (S11) of the input port below −15 dB over a broad bandwidth. The second harmonic (TEM mode) passes through the region between the diodes and the input back-short, and then couples into the output probe by the matching circuit. Due to the orthogonality of the two modes, the input and output of the diode cell are highly isolated. Hence, there is no need to design an extra filter. As shown in Figure 4, a ladder structure is added in the diode cell, which can improve the matching impedance to expand the bandwidth. Finally, an output structure needs to be designed that mainly includes microstrip-waveguide transition and a direct current (DC) filter. Specifically, a probe located in the transition circuit couples the second harmonic to the standard output waveguide. In addition, the filter provides a way to feed the DC bias to the diode cell.

The nonlinear design does not begin until the above-mentioned linear analysis steps have finished. The generated S matrix files extracted from each part of the doubler are imported to the ADS circuit, and the nonlinear characteristic of the diode is also added into the circuit. The optimization procedure based on the harmonic balance (HB) analysis is achieved to realize a maximum output power in a wide bandwidth by adjusting the length of discrete matching elements (L1–L7), which are treated as variable parameters in the simulation. Actually, by tuning the length, the input power is maximized to the diode cell, and reduces the reflection at the desired harmonic. It is noted that the source impedance and load impedance are set to be equal to the characteristic impedances of the input waveguide (WR8) and output waveguide (WR4), respectively.

Regarding the tripler, it is a split-block waveguide design, with a monolithic integrated circuit based on a 25 μm thin gallium arsenide substrate which is mounted in the channel between the input and output waveguide. To maximize the conversion efficiency, an unbalanced structure with a pair of diode chips in parallel is adopted, as shown in Figure 3b. Although the bandwidth of this scheme is relatively narrow, it is suitable for designing a high-efficiency tripler. 

**Figure 5:**
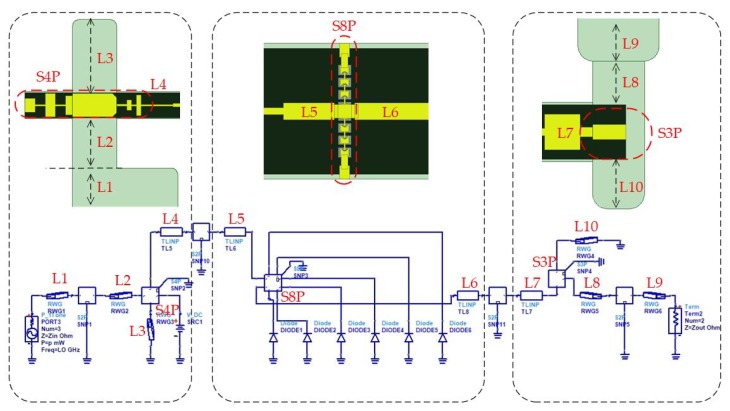
Design of the 215 GHz monolithic integrated tripler based on the field-circuit method (L1 = 0.5 mm, L2 = 0.725 mm, L3 = 1.06 mm, L4 = 0.47 mm, L5 = 0.15 mm, L6 = 0.33 mm, L7 = 0.24 mm, L8 = 0.28 mm, L9 = 0.2 mm, and L10 = 0.32 mm).

The equivalent linear and nonlinear models of the tripler are shown in Figure 5. The passive part of the tripler is divided into three parts as depicted in the figure, i.e., the input circuit, output circuit, and diode cell. The input circuit, as well as the output circuit, consist of the waveguide-microstrip transition (input and output probe) and matching circuit. In addition, the input circuit also includes a low pass DC bias filter and a fundamental frequency low pass filter. 

The fundamental frequency is coupled to the main transmission line via the input E-plane probe and passes through the filter to the diode cell. Generally, the length of the reduced-height waveguide and the location of the input back-short are optimized to achieve the return loss of the input port below −20 dB and over 70~75 GHz bands. Meanwhile, the DC filter plays a role in preventing the fundamental frequency from leaking into the bias circuit. The main body of the output circuit is also an E-probe, in conjunction with an output back-short and an output reduced-height waveguide. The probe located in the output circuit couples the third harmonic to the standard output waveguide. Hence the output structure design is identified when the transmission coefficient is above −0.5 dB (S21) at 210~220 GHz.

After the linear electromagnetic structure simulation is done, the S parameters of each part with discrete matching elements (L1–L10) are exported to ADS so that the nonlinear HB analysis can be carried out. The goal of the above process is to obtain the desired conversion efficiency by adjusting the matching elements.

Finally, for optimization as a whole, the tripler model can be regarded as a nine-port network, which includes the input waveguide port, output waveguide port, DC filter port, and six lumped ports for Schottky diodes.

## 4. Results

**Figure 6:**
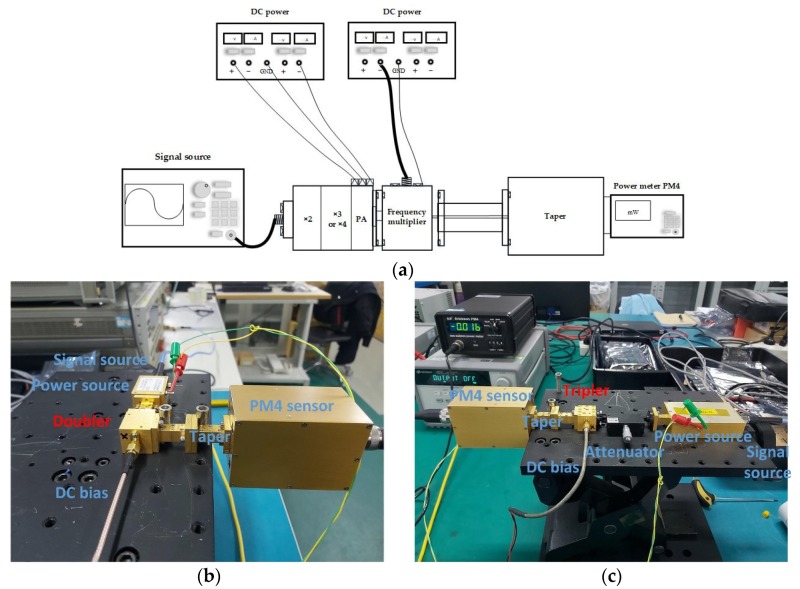
(**a**) Test bench and measurement diagram of the frequency multipliers; (**b**) Photo of test site for the the 200 GHz doubler; (**c**) Photo of test site for the 215 GHz tripler.

The block diagram of the measurement setup is illustrated in Figure 6a. Actually, the power source used to drive the 200 GHz doubler is a W band, active 8× frequency multiplier, which has an input frequency of 10.7 to 13.3 GHz with a typical output +10 dBm from 86 to 106 GHz. In addition, the input signal is provided by an Agilent analog signal generator E8257D (Keysight technologies, Inc., Santa Rosa, CA, USAManufacturer, City, Country), and the output power of the 200 GHz doubler is measured using a Virginia Diodes, Inc. (VDI, Charlottesville, USA) Erickson PM4 power meter. A photo of the 200 GHz doubler test bench is shown in the Figure 6b. 

**Figure 7:**
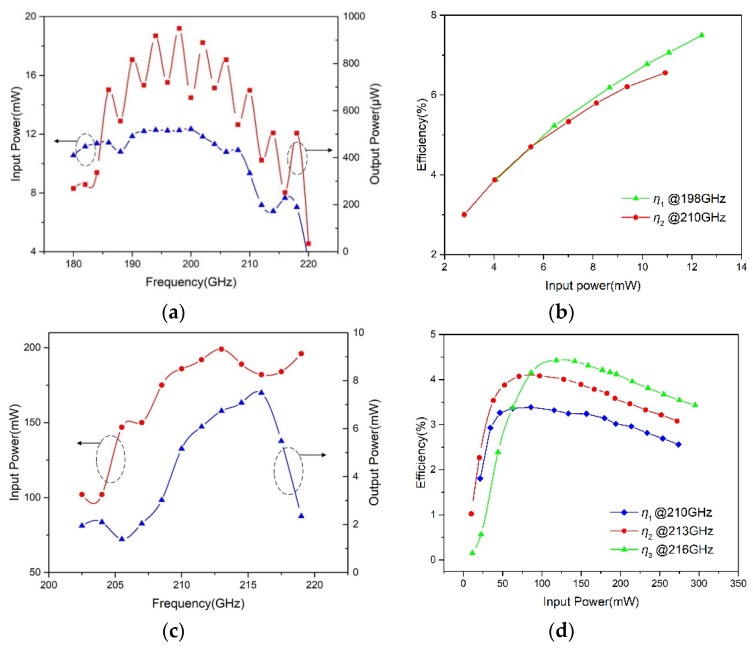
Measured results of frequency multipliers. (**a**) Measured input and output power of the 200 GHz doubler as a function of frequency; (**b**) Measured efficiency of the 200 GHz doubler as a function of input power at fixed frequency; (**c**) Measured input and output power of the 215 GHz tripler as a function of frequency; (**d**) Measured efficiency of 215 GHz tripler as a function of input power at fixed frequency.

The measured results of the monolithic integrated 200 GHz doubler are shown in Figure 7. From Figure 7a, it is easily observed that the measured output power of the doubler is more than 250 μW at 180~218 GHz and the maximum output power is about 950 μW at 198 GHz. Due to the corresponding input power in the range of 7 to 12.3 mW, the typical efficiency of the 200 GHz doubler is about 6%. It can be seen from the graph some fluctuations exist in the band because of broadband design.

In fact, the optimum input power of the 200 GHz doubler is 60 mW in the design. However, it is difficult to obtain the highest efficiency because it lacks a drive source with enough power. Therefore, another measurement is done to explain the relationship of the efficiency and input power, as shown in Figure 7b. The result shows that the efficiency increases with the input power, and the maximum conversion efficiency is about 7.5% at 198 GHz. Actually, the efficiency would continue to increase based on a trend derived from the graph if there is a higher input power. 

The photo of 215 GHz tripler test bench is shown in the Figure 6c. The drive power generating the signal over 300 mW at 68–77 GHz, mainly includes a sextupler and a power amplifier. The sextupler employs commercially available Gallium Arsenide chip fabricated by UMS company (United Monolithic Semiconductors (UMS), Villebon-sur-Yvette, France), and the power amplifier uses the MMIC chip, fabricated by Hittite microwave corporation (Hittite, Norwood, MA, USA). An attenuator is added between the source and the tripler to control the input power. The output power of the 215 GHz tripler is measured using a PM4 power meter. Moreover, thanks to the unbalanced structure, a DC supply connected to the SMA port can bias the varactors.

Under the condition of external reverse bias of approximately 4.5 V, the measured input and output power of the tripler are plotted in Figure 7c. It can be found that the output power is more than 5 mW at 210~218 GHz. In this test, the maximum is about 7.3 mW at 216 GHz when driven with 194 mW of input power, and the corresponding conversion efficiency is close to 3.8%. Due to the sufficient power, it is necessary to study the relationship between efficiency and input power. Corresponding results are shown in Figure 7d. When the frequency is fixed, the efficiency changes along with an increase of input power and reaches the peak value. Then, the efficiency drops as the input power increases continuously because a saturation phenomenon occurs, and hence the effective series resistance increases rapidly with higher power levels [28,29]. In this test, the highest efficiency is close to 4.5% and the maximum output power is more than 10 mW, when driven with 295 mW at 216 GHz. In addition, the best value of input power changes with the different frequency. 

Limited by the semiconductor process, there are not many domestic reports on the design of terahertz frequency multipliers based on a monolithic integrated circuit. Table 2 illustrates a simple comparison of some reported frequency multipliers. Among them, VDI is one of the best research institutions in the terahertz solid-state circuit field, and some advanced technology such as frameless membrane has been developed. It can be found that the performance of the frequency multipliers presented in this paper, has a leading position in China. Despite a gap in the semiconductor process, the performance is close to the level achieved by VDI due to a global design method.

## 5. Conclusions

In summary, a 200 GHz broadband MIC doubler and a 215 GHz narrowband MIC tripler with 3-dB fractional bandwidth up to 12% and 4%, respectively, have been designed in this paper. The measured results indicated that, on the one hand, the output power of the doubler is more than 250 μW at 180~218 GHz, and the typical efficiency is about 6%. On the other hand, the measured output power of the tripler is above 5 mW at 210~218 GHz. Actually, the output power could exceed 10 mW at 216 GHz. This research focuses on the terahertz monolithic integrated circuit and solves a series of key problems such as design of a terahertz device, process realization, etc. It provides a technical proposal to overcome the difficulties that exist in the hybrid integrated circuit and makes it possible for multipliers to work at a higher frequency. In addition, TMIC also improves the stability and consistency.

## Figures and Tables

**Table 1:** The simulated and measured parameters of the Schottky diodes used in the design of the frequency multipliers. In the table, s and m represent simulated and measured results, respectively.

Diameter/μm	R_S_/Ω		C_j0_/fF		n		I_S_/E-13 A	
	s	m	s	m	s	m	s	m
3	7.1	8.4	11.0	12.1	1.19	1.15	0.97	1.26
5	4.8	5.0	29.6	29.5	1.19	1.19	1.95	2.81

**Table 2:** Performance comparison of the frequency multipliers based on TMIC.

References	Technology	Frequency (GHz)	Max Output Power (mW)	Efficiency
[30] from UESTC (CHN)	×2 TMIC	211.92–214.8	0.53	0.5% (Max)
[31] from UESTC (CHN)	×3 TMIC	330–500	0.194	2% (Max)
[32] from VDI (USA)	×2 TMIC	140–220	–	7.5% (typical)
	×3 TMIC	140–220	–	3% (typical)
This paper	×2 TMIC	180–218	0.95	7.5% (Max)
	×3 TMIC	210–218	10.1	4.5% (Max)
